# Horizontal Gene Transfer of Functional Type VI Killing Genes by Natural Transformation

**DOI:** 10.1128/mBio.00654-17

**Published:** 2017-07-25

**Authors:** Jacob Thomas, Samit S. Watve, William C. Ratcliff, Brian K. Hammer

**Affiliations:** School of the Biological Sciences, Georgia Institute of Technology, Atlanta, Georgia, USA; Princeton University

**Keywords:** type VI secretion, *Vibrio cholerae*, genetics, horizontal gene transfer, natural transformation systems

## Abstract

Horizontal gene transfer (HGT) can have profound effects on bacterial evolution by allowing individuals to rapidly acquire adaptive traits that shape their strategies for competition. One strategy for intermicrobial antagonism often used by *Proteobacteria* is the genetically encoded contact-dependent type VI secretion system (T6SS), a weapon used to kill heteroclonal neighbors by direct injection of toxic effectors. Here, we experimentally demonstrate that *Vibrio cholerae* can acquire new T6SS effector genes via horizontal transfer and utilize them to kill neighboring cells. Replacement of one or more parental alleles with novel effectors allows the recombinant strain to dramatically outcompete its parent. Using spatially explicit modeling, we examine how this process could affect the ecology and evolution of surface-attached microbial populations. HGT of T6SS effector-immunity pairs is risky: transformation brings a cell into conflict with its former clone mates but can be adaptive when superior T6SS alleles are acquired. More generally, we find that these costs and benefits are not symmetric and that high rates of HGT can act as a hedge against competitors with unpredictable T6SS efficacy. We conclude that antagonism and horizontal transfer drive successive rounds of weapon optimization and selective sweeps, dynamically shaping the composition of microbial communities.

## IMPORTANCE

The contact-dependent type VI secretion system (T6SS) is frequently used by *Proteobacteria* to kill adjacent competitors. While DNA released by T6 killing can be horizontally acquired, it remains untested whether T6 genes themselves can be horizontally acquired and then utilized to compete with neighboring cells. Using naturally transformable *Vibrio cholerae*, we provide the first direct empirical support for the hypothesis that T6 genes are exchanged horizontally (e.g., from dead competitors) and functionally deployed to compete with neighboring cells. Using computational simulations, we also demonstrate that high rates of HGT can be adaptive, allowing *V. cholerae* to improve upon existing T6 weaponry and survive direct encounters with otherwise superior competitors. We anticipate that our evolutionary results are of broad microbiological relevance, applying to many bacteria capable of HGT that utilize the T6SS or similar antagonistic systems, and highlight the profound impact of HGT in shaping microbial community structure.

## INTRODUCTION

Horizontal gene transfer (HGT) by plasmid conjugation, viral transduction, and natural transformation plays a fundamental role in the evolution of bacteria and archaea and also in plants and other eukaryotes (1). Genomic analyses implicate HGT as a major factor responsible for the mosaic genomes of many bacteria, including the waterborne microbe *Vibrio cholerae*, which often carries the cholera toxin (CTX)-encoding prophage responsible for major cholera outbreaks in Haiti and regions of endemicity (2–5). *V. cholerae* isolates from aquatic environments are invariably nontoxigenic (CTX^−^) and commonly associated with chitinous surfaces like shells of crabs or zooplankton that may promote transmission (6).

All sequenced isolates of *V. cholerae* encode a type VI secretion system (T6SS) weapon that can deliver toxic effector proteins directly into grazing predators, diverse proteobacteria, and other *V. cholerae* bacteria that lack cognate immunity proteins (reviewed in references 7 to 11). The majority of nonclinical isolates express the T6SS constitutively, in the absence of chitin, likely as a competitive strategy in complex microbial communities of environmental biofilms (12, 13). In contrast, the majority of toxigenic clinical *V. cholerae* isolates engage in contact-dependent T6 killing only when triggered by the presence of chitin (14, 15), perhaps downregulating this activity in a host (9). Interestingly, chitin can also induce natural transformation in clinical isolates, which can promote HGT events due to lysis of adjacent nonimmune neighbors (14). Genome analyses of *V. cholerae* and other *Vibrio* species suggest that T6 loci encoding distinct effectors and adjacent cognate immunity factors, yet flanked by highly conserved sequence, may themselves be horizontally acquired by homologous recombination (16–18). Furthermore, it was hypothesized, but not experimentally tested, that HGT of distinct effector-immunity pairs could enable populations to diversify by generating individuals compatible with their competitor, yet susceptible to kin (16, 18). Whether HGT of novel T6 genes is adaptive in communities where kin and nonkin are in close proximity is unclear. Most clinical *V. cholerae* strains, which constitute the majority of sequenced and characterized isolates, carry identical T6 loci, thus hampering efforts to address this challenge.

We recently demonstrated that two sequenced *V. cholerae* strains with two syntenic T6 loci encoding distinct effector-immunity pairs behaved as “mutual killers” that precipitated a phase separation when cocultured (19). One strain is derived from clinical reference isolate C6706, which is induced by chitin to simultaneously express both the DNA uptake apparatus and the T6SS. The other is a recently sequenced environmental isolate 692-79 (20) which is defective at DNA uptake and yet is T6SS proficient. Here, we show that coculturing of these two strains in the presence of chitin induces unidirectional horizontal transfer of novel T6 effector-immunity pairs to the clinical isolate by homologous recombination. Transformants are simultaneously protected from attack by the environmental donor and susceptible to attack by former siblings. We modeled this process to gain insight into the evolutionary and ecological dynamics of T6-mediated competition. The fitness costs and benefits from acquisition and replacement of distinct T6SS effectors are contextual and should depend on the spatial structure of the population, the relative efficacy of T6SS effectors, and the rate of horizontal transfer. These results demonstrate that members of microbial communities can continuously adapt to diverse competitors by rapid acquisition of new molecular weaponry via horizontal transfer.

## RESULTS

**FIG 1:**
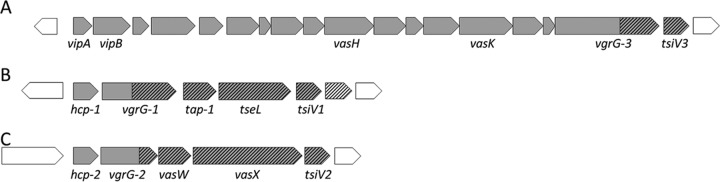
Organization of T6SS operons in *Vibrio cholerae* C6706. T6SS genes, shaded in gray, are organized into one large cluster (A) and two auxiliary clusters, Aux1 (B) and Aux2 (C). Hatched regions are variable compared to other sequenced *V. cholerae* isolates. The first gene of each of the auxiliary clusters encodes the major subunit of the protein tube, Hcp. Genes encoding effector and immunity proteins are *vgrG-3* and *tsiV3* (main cluster), *tseL* and *tsiV1* (Aux1), and *vasX* and *tsiV2* (Aux2), respectively.

The core T6SS components of all sequenced *V. cholerae* strains are encoded in three operons—one main cluster and two auxiliary clusters, Aux1 and Aux2 (Fig. 1), while additional T6SS loci that may encode new effector-immunity pairs thought to be dispensable for assembly of the T6SS spike are occasionally present (18, 21). Each operon terminates in a gene encoding a toxic effector, immediately followed by its cognate immunity gene. In the toxigenic C6706 strain, the main cluster effector protein, VgrG-3, is a “specialized” effector that forms a physical component of the puncturing spike tip, while the Aux1 and Aux2 effectors are “cargo” effectors that each require a specific Tap (T6SS adaptor protein) or PAAR (proline-alanine-alanine-arginine) repeat domain protein as a chaperone for assembly with its cognate VgrG spike protein (8, 22). Aux1 and -2 operons of all sequenced *V. cholerae* strains have conserved DNA sequences flanking a variable region of differing percent GC content that encodes the effector-immunity pair, Tap, and C terminus of VgrG (18). The main T6SS cluster is entirely conserved between *V. cholerae* strains except for a short VgrG-3 C terminus and a portion of the immunity gene.

As genomic analyses predict that transfer of entire T6SS operons frequently occurs in nature (16, 18), we tested this experimentally with toxigenic reference strain C6706, which is induced by chitin to become naturally transformable, and a nontransformable environmental isolate, 692-79 (12). At the Aux1 locus, both strains have distinct phospholipase effectors: TseL in C6706 and a phospholipase similar to Tle1 of *Pseudomonas aeruginosa* in 692-79 (23, 24). The Aux2 operons of the two strains encode effectors with entirely different activities: a VasX pore-forming protein in C6706 and a LysM domain-containing effector that potentially targets peptidoglycan in 692-79 (25, 26). We recently demonstrated that the distinct T6 activities of these strains allowed them to engage in mutually antagonistic T6S killing (19). We therefore predicted that horizontal transfer of T6SS genes via natural transformation from a 692-79 donor to a C6706 recipient would generate transformants with T6SS profiles distinct from both donor and recipient strains. To test whether we could observe C6706 transformants that acquired the Aux alleles of the 692-79 donor, we introduced antibiotic resistance cassettes by recombination directly downstream of the Aux1 or Aux2 immunity genes in the 692-79 strain. To avoid challenges interpreting exchange of core and regulatory components, we did not design a system to measure exchange of large cluster components. When the two strains were cocultured on chitin tiles submerged in artificial seawater (ASW), C6706 acquired the entire Aux1 or Aux2 operon of 692-79 by natural transformation at frequencies of ~1 × 10^−6^, while a nontransformable C6706 mutant lacking the *comEA* gene did not (see Fig. S1 in the supplemental material). Acquisition of each T6SS operon was confirmed by PCR analysis. C6706 cultured with chitin was able to acquire both Aux1 and Aux2 T6SS alleles either from genomic DNA derived from 692-79 or by coculture with the donor strain (Fig. S1), and successive rounds of chitin-induced transformation also generated a C6706 transformant with both Aux1 as well as Aux2 allele replacements. Since acquisition by homologous recombination of a new T6SS operon is accompanied by a simultaneous loss of the original alleles, we initially expected that transformants would be selected against due to T6S killing by neighboring former clone mates. However, T6SS inactivation by deletion of *vasK* encoding an assembly protein (27) had modest effects on acquisition frequency of new T6SS operons either when using genomic donor DNA or in coculture (Fig. S1). We suspect that this is likely because the substrate-adherent cell-cell contact required for T6SS-mediated killing is not consistently maintained under the seawater conditions used in our transformation assays.

10.1128/mBio.00654-17.1 FIG S1  T6SS operons can be acquired by natural transformation. (A) *V. cholerae* C6706 and its Δ*vasK* (T6SS^−^) or Δ*comEA* (Com^−^) nontransformable derivatives were used as recipients in standard transformation assays in artificial seawater with induction by chitin, using 692-79 donor DNA marked with antibiotic resistance at the Aux1 (A1, black bars) or Aux2 (A2, white bars) locus. Inheritance of the donor T6SS Aux1 or Aux2 operon in resistant transformants was verified by colony PCR. (B) Recipients were cocultured with donor strains derived from 692-79 that were marked with antibiotic resistance at the Aux1 (A1, black bars) or Aux2 (A2, white bars) locus in artificial seawater supplemented with chitin. Donor strains were also inactivated for T6SS (A1 Δ*vasK*, dark gray bars, and A2 Δ*vasK*, light gray bars). Bars denote means ± standard deviations (*n* = 6 biological replicates). Download FIG S1, TIF file, 0.1 MB. Copyright © 2017 Thomas et al. 2017 Thomas et al. This content is distributed under the terms of the Creative Commons Attribution 4.0 International license.

**FIG 2:**
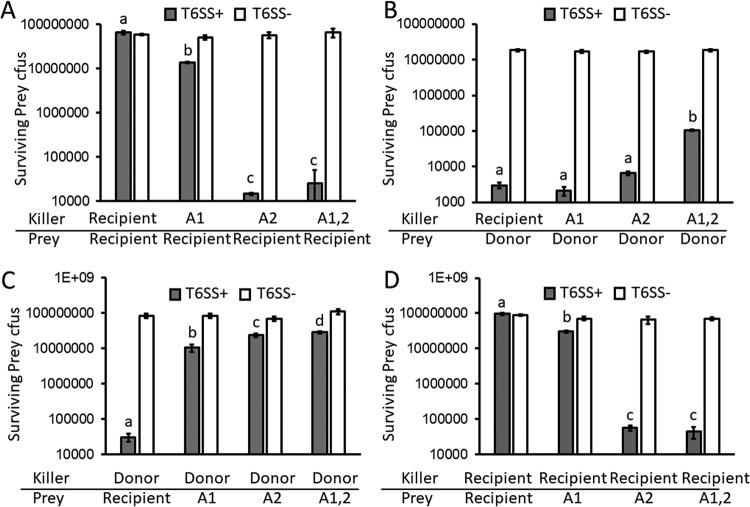
Horizontally acquired T6SS effectors and immunity proteins are functional in the recipient strain. “Killer” strains and T6SS^−^ Δ*vasK* “prey” strains were mixed at a 10:1 ratio and incubated at 37°C for 3 h on membrane filters, and surviving prey CFU were counted on selective medium. Killer strains were either T6SS^+^ (gray bars) or T6SS^−^ (Δ*vasK*, white bars). Strains used were C6706 (recipient), 692-79 (donor), and C6706 derivatives in which either Aux1 (A1), Aux2 (A2), or both clusters (A1,2) were replaced by those of 692-79. Bars denote means ± standard deviations (*n* = 4 biological replicates, one representative experiment shown of two performed). T6SS^+^ genotypes with different letters (a, b, c, d) exhibited mean survival different from those of other T6SS^+^ genotypes (*P* < 0.05, ANOVA and Tukey’s HSD) within the same experiment (panel).

Recombination of each entire Aux cluster in the C6706 transformants was verified by sequencing the effector and immunity genes and a stretch of DNA 2 kb upstream of the start codon of the first gene in each operon. Sequencing confirmed acquisition of both the effector and immunity genes of the 692-79 donor, but newly acquired T6SS effectors must be expressed, properly folded, and loaded onto the T6SS complex encoded by the recipient strain if they are to be functional. Indeed, we found that the C6706 transformants with the Aux1 T6SS operon from 692-79 (which we refer to here as A1), the Aux2 operon (A2), and both operons (A1,2) are able to kill C6706 to an increasing degree in standard 3-h T6 killing assays (*F*_3,12_ = 241.5, *P* < 0.0001 [Fig. 2A]; differences in treatments for T6SS^+^ experiments in Fig. 2 were assessed via analysis of variance [ANOVA] and Tukey’s honestly significant difference [HSD]). Acquisition of either cluster also diminished the ability of transformants to kill 692-79; however, replacement of both clusters did not completely abrogate killing of 692-79 (*F*_3,12_ = 983.2, *P* < 0.0001 [Fig. 2B]). We predicted that the residual killing activity might be due to expression of the main cluster VgrG-3 of C6706, which was not replaced in these experiments. Indeed, deletion of *vgrG-3* in the double (A1,2) transformant completely abolished killing of the donor 692-79 (Fig. S2). Replacement of T6SS clusters also changed the immunity profiles of each strain: a single or double Aux cluster replacement protected the transformant from killing by the 692-79 donor (*F*_3,12_ = 126.5, *P* < 0.0001 [Fig. 2C]) but made the strains susceptible to killing by the C6706 recipient (parent), with the largest effect seen in the Aux1,2 transformant (*F*_3,12_ = 446.5, *P* < 0.0001 [Fig. 2D]).

10.1128/mBio.00654-17.2 FIG S2  VgrG-3 is responsible for residual T6SS-killing activity of the T6SS Aux1 and -2 replacement strain toward its donor. “Killer” strains and T6SS^−^ Δ*vasK* “prey” strains were mixed at a 10:1 ratio and incubated at 37°C for 3 h on membrane filters, and surviving prey CFU were counted on selective medium. Killer strains were either T6SS^+^ (gray bars) or T6SS^−^ (Δ*vasK*, white bars) and had a functional VgrG-3 main cluster effector (A) or were deleted for *vgrG-3* (B) Strains used were C6706 (recipient), 692-79 (donor), and C6706 derivatives in which either Aux1 (A1), Aux2 (A2), or both clusters (A1,2) were replaced by those of 692-79. Bars denote means ± standard deviations (*n* = 4 biological replicates). Download FIG S2, TIF file, 0.1 MB. Copyright © 2017 Thomas et al. 2017 Thomas et al. This content is distributed under the terms of the Creative Commons Attribution 4.0 International license.

**FIG 3:**
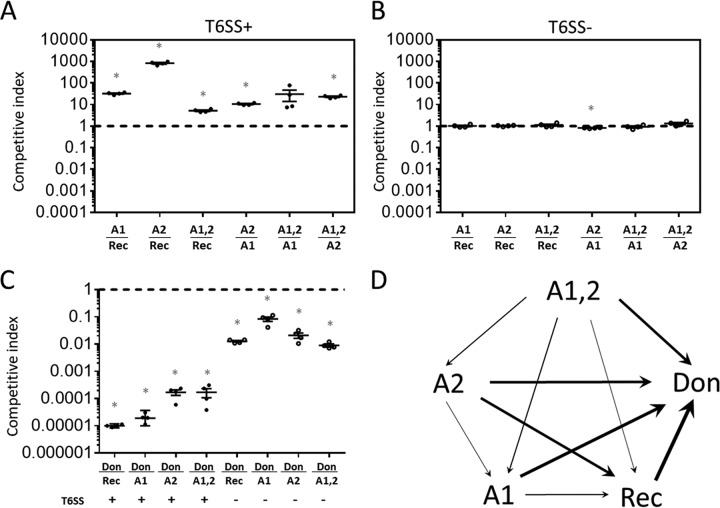
Acquisition of T6SS effectors from a donor (692-79) is beneficial to the recipient (C6706). (A to C) Pairwise competitions were set up between the recipient strain C6706 (Rec), the donor 692-79 (Don), and C6706 derivatives in which either Aux1 (A1), Aux2 (A2), or both clusters (A1,2) were replaced by those of 692-79. Strains were mixed at a 1:1 ratio and incubated on solid agar in a 12-well plate at 37°C for 24 h, and survivors of each strain were counted on selective medium. Both strains were T6SS^+^ (A and C, closed circles) or T6SS^−^ (Δ*vasK*, B and C, open circles). Competitive indices are measured as the final ratio of survivors divided by the initial ratio of inoculation. Horizontal lines in each cluster indicate geometric means of competitive indices. (D) The competitive hierarchy of strains analyzed in panels A to C is represented with arrows proceeding from the more fit competitor toward the less fit competitor. The line weight of each arrow is proportional to the log_10_ of the geometric mean of the competitive indices of the (more fit/less fit) competitor. Geometric mean and geometric standard deviation are represented for each cluster in panels A to C (*n* = 4 biological replicates, one representative experiment shown of two performed). Asterisks denote a competitive index significantly (*P* < 0.05) different from 1 (Bonferroni-corrected two-tailed one-sample *t* tests).

A previous study demonstrated that the effector-immunity alleles carried by *Vibrio cholerae* V52 (and C6706) were the most potent in pairwise competition experiments among the different allele combinations tested (18). To examine the relative fitness of each T6SS transformant with respect to the recipient (parent) and donor, we performed pairwise 24-h competition experiments (Fig. 3). Acquisition of each T6SS cluster (with the exception of Aux1,2 versus Aux1) conferred a significant advantage to the transformant relative to the recipient (Fig. 3A). Statistical significance was established via two-tailed one-sample *t* tests with a Bonferroni correction for multiple comparisons. All tests with T6SS^+^ genotypes were significant (*P* < 0.01, Fig. 3A and C), with the exception of Aux1,2 versus Aux1 (Fig. 3A), which had a postcorrection *P* value of 0.076. In every other case, transformants carrying an Aux1 (A1), Aux2 (A2), or both Aux1 and 2 (A1,2) replacement had increased fitness over both recipient and donor (Fig. 3A), with only marginal competitive differences observed in T6SS^−^ controls (Fig. 3B). The A1,2 transformant has a competitive advantage over every other strain tested, suggesting that when 692-79 is the donor, acquisition of T6SS alleles by HGT is always beneficial to the C6706 recipient. However, the donor has a large T6SS-independent fitness defect compared to C6706 and its derivatives (Fig. 3C) and represents a potentially rare example of a poor competitor that carries superior weapons. To verify the competitive hierarchy that we observed in two-partner competitions (Fig. 3D), we cocultured C6706 and each transformant (A1, A2, and A1,2) in an initial 1:1:1:1 ratio for 5 days with back dilution and repeat passaging every 24 h (Fig. S3). We found that the A1,2 transformant dominated the mixed culture within 24 h and that the relative representation of the other strains was in agreement with the hierarchy observed in two-partner competitions (Fig. 3). Thus, HGT of T6SS allows C6706 to rapidly improve its repertoire of T6SS weaponry.

10.1128/mBio.00654-17.3 FIG S3  Horizontally acquired T6SS alleles are beneficial in long-term competition assays. Derivatives of C6706 were mixed in a 1:1:1:1 initial ratio, allowed to compete on nonselective solid agar for 24 h at 37°C, and resuspended, and surviving CFU were counted on selective medium. The suspension was repeat passaged on solid agar for 5 days. Strains used were C6706 (Rec), 692-79 (donor), and C6706 derivatives in which either Aux1 (A1), Aux2 (A2), or both clusters (A1,2) were replaced by those of 692-79. All strains were either T6SS^+^ (A) or T6SS^−^ (B). Symbols denote mean ± standard deviation (*n* = 4 biological replicates). Download FIG S3, TIF file, 0.1 MB. Copyright © 2017 Thomas et al. 2017 Thomas et al. This content is distributed under the terms of the Creative Commons Attribution 4.0 International license.

**FIG 4:**
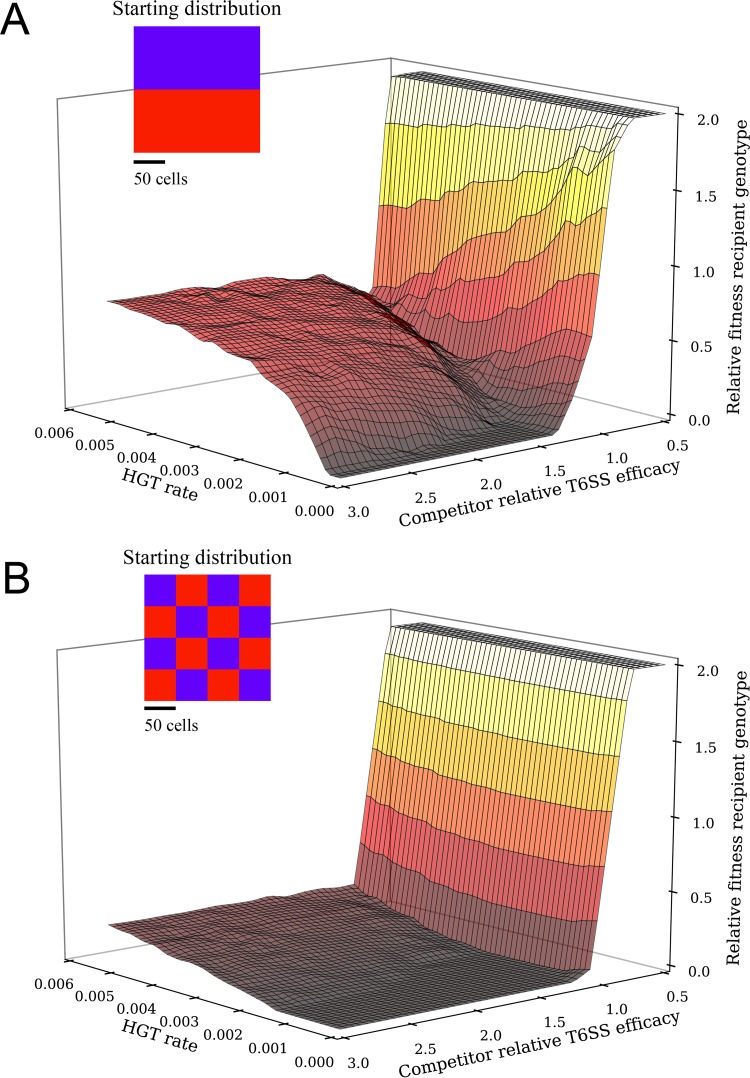
Costs and benefits of horizontally acquiring a competitor’s T6SS. In this model of competition between two bacterial strains, one (the “recipient”) was capable of integrating T6SS alleles from the other (the “competitor”). High rates of HGT were adaptive only when the recipient strain faced a competitor with superior T6SS alleles. For plotting purposes, relative fitness of >2 was clipped. Simulations were run for 15,000 time steps. Time-lapse videos of these fitness landscapes are shown in Movies S2 and S3.

Our experimental results highlight a specific scenario of transfer of T6SS clusters. In order to derive general principles for how natural selection might act on the HGT of T6SS clusters, we extended a model that we previously developed to consider growth and competition on a two-dimensional lattice between any two mutually antagonistic strains (19). In our simulation, each strain possessed three distinct T6SS alleles. The “recipient” strain was capable of sequentially replacing its T6 effectors with those obtained from the “competitor” strain (see Materials and Methods for model details). As in our experiments, the recipient was capable of HGT, but the donor was not. We observed that HGT can have a profound impact on T6SS-mediated competition dynamics. When the recipient possesses inferior T6SS effectors, HGT events precipitate a series of selective sweeps resulting in transformants that have completely replaced their T6SS alleles with those obtained from the superior competitor strain (Movie S1). As a result, HGT allows for competitive rescue of a strain that encodes an inferior set of T6SS alleles (relative T6SS efficiency, <1). More generally, the consequences of HGT on the fitness of the recipient genotype depend on how its T6SS loci compare to those of its competitor and the degree to which the population is spatially structured (Fig. 4A relative to 4B). Higher HGT rates are favored when a competitor possesses superior T6SS alleles but are costly when the competitor’s T6SS alleles are inferior (Fig. 4). The benefits of HGT are far greater when the population is highly structured with each strain spatially separated (e.g., Fig. 4A), since T6 cluster replacement via HGT takes time. When the population is highly structured, the superior competitor takes more time to displace the recipient, which allows sufficient time for HGT and allelic replacement of T6SS clusters. The fitness valley in Fig. 4A occurs when horizontally acquired T6SS alleles are slightly better than those possessed by the recipient, allowing them to kill their clone mates, but not enough to escape their geometric disadvantage and spread. As a result, HGT drives high rates of intraclonal conflict, reducing their relative fitness.

10.1128/mBio.00654-17.5 MOVIE S1 Visualization of the dynamics of HGT and T6SS. This simulation examines HGT under favorable conditions: the population is initially highly structured and the competitor possesses more efficacious T6SS alleles (3-fold better than the recipient). Recipient cells that take up one allele from the blue competitor are shown in green, those that have taken up two alleles are shown in orange, and those that have swapped out all three of their T6SS alleles are shown in yellow. Transformants possess a strong within-patch selective advantage, leading to repeated waves of HGT and T6SS-mediated sweeps until all of the recipient cells possess their competitors’ T6SS effector alleles (yellow). In this simulation, each cell had a 0.0005 probability of undergoing HGT per time step. Download MOVIE S1, AVI file, 7.8 MB. Copyright © 2017 Thomas et al. 2017 Thomas et al. This content is distributed under the terms of the Creative Commons Attribution 4.0 International license.

**FIG 5:**
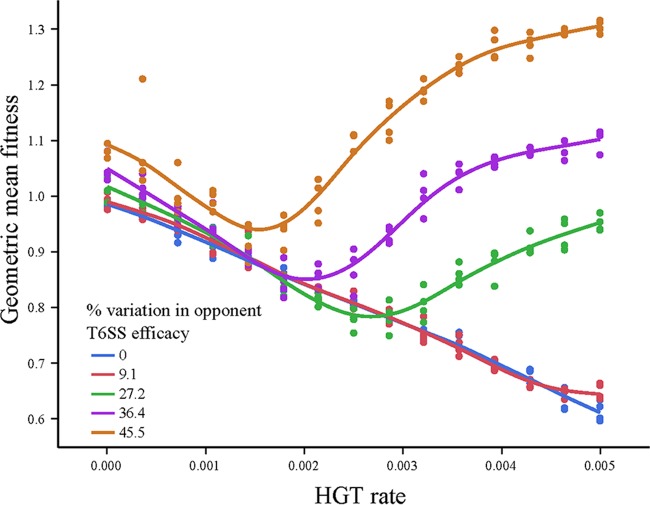
Horizontal gene transfer serves as a hedge against unpredictable competitors. To examine effects of variability in competitor T6SS efficacy on selection on HGT rate, the geometric mean fitness of the recipient genotype was determined across pairs of simulations, in which recipients faced competitors with increasingly high variation in T6SS efficacy (±0, 9.1, 27.2, 36.4, and 45.5% from its own T6SS).

T6SS-mediated conflict is inherently risky, as competitors vary widely in their relative killing efficacy (18). While HGT may allow a strain with inferior T6SS effectors to survive an interaction with a superior competitor in which it might otherwise be eradicated, HGT may also be costly, as rare transformants that acquire foreign T6SS loci immediately turn on adjacent former clone mates and are usually killed (Movie S1). While it is clear that incorporating inferior T6SS effectors will be costly, this is a separate cost from intrastrain genetic conflict, which occurs even when superior T6SS effectors are obtained horizontally. To estimate the fitness consequences of HGT-mediated intrastrain conflict, we simulated competition when mutually antagonistic recipients and competitors had distinct T6SS clusters that nonetheless killed with equal efficacy and then varied the HGT rate (Fig. 5, blue points). We measured the geometric mean fitness, as this best predicts long-term changes in the frequency of each T6 effector and is especially useful for taking variance in fitness across generations into account (28). Within-strain conflict, which can be estimated by the regression of fitness on HGT rate when partners do not vary (blue points; *y* = −74.2*x* + 0.991, *r*^2^ = 0.99), was very costly, reducing fitness by 40% when the per-cell probability of HGT per time point was 0.005.

The fact that HGT incurs significant costs due to intrastrain conflict and benefits via survival when facing a superior competitor raises the possibility that it may act as a hedge against uncertainty in the T6SS efficacy of future opponents. We examined this hypothesis by calculating a recipient strain’s geometric mean fitness across a pair of interactions in which it faced a competitor with symmetrically varying T6SS efficacy (there was thus no overall bias in favor of either strain) and across a range (0 to 0.005) of HGT. A geometric mean fitness above 1 indicates that HGT is adaptive. When the two strains have T6SSs with similar efficacies, the costs of HGT (intrastrain conflict) exceed its benefits and selection acts against HGT (Fig. 5). However, when competitor quality is highly variable, HGT can be adaptive, with selection favoring very high rates of transformation (Fig. 5). The valley-shaped distribution of fitness for the high-variance treatments is a consequence of the geometry of competition: when HGT rates are low, transformants are surrounded by former clone mates that can now kill them via T6SS. Isolated transformants have a low probably of surviving killing attempts by their neighbors, even if they have moderately superior T6SS alleles. Higher rates of HGT lead to rare but important multiple transformation events where two or more adjacent cells take up a competitor’s allele, reducing their geometric disadvantage and dramatically increasing the probability that they will precipitate a selective sweep. Note that even when HGT equals 0, variation in competitor T6SS increases the recipient’s fitness (Fig. 5). This reflects the cost of variability on the competitor’s fitness (or, inversely, the relative benefit that the recipient experiences by not varying), a well-known phenomenon in evolutionary ecology (28). Thus, high rates of HGT were strongly beneficial when the competitor’s T6SS efficacy was more variable, as the increased survival in the face of a superior competitor more than made up for HGT’s costs.

## DISCUSSION

We have demonstrated that toxigenic bacteria can acquire T6SS effector-immunity pairs from nontoxigenic neighbors via natural transformation, consistent with the hypothesis that this frequently occurs in the environment (16, 18). Once acquired, the novel T6SS effector-immunity proteins are functional and can contribute toward T6SS-mediated antagonism against neighboring cells as well as provide immunity against T6SS attacks by neighbors. Interestingly, transformants that undergo successive HGT of T6SS effector-immunity pairs have increased fitness relative to either parental strain in direct competition experiments, reaffirming the fundamental role of HGT in shaping bacterial ecology and evolution. To provide ecological and evolutionary context for these results, we used an agent-based model to explore how HGT of T6SS could affect spatial patterning in surface-attached populations and generate hypotheses for how key parameters (i.e., population spatial structure, rate of HGT, relative efficacy of T6SS alleles between competitor strains, and variance in competitor T6SS efficacy) might affect bacterial fitness. Our modeling reveals that HGT can be quite costly: a cell runs the risk of incorporating an inferior T6SS allele, and every transformant must overcome the geometric disadvantage of being surrounded by former clone mates to which it is no longer fully immune. Still, these costs are more than compensated for when an HGT^+^ strain is facing a competitor with far superior T6SS alleles. Indeed, we show that variability in opponent T6SS allele quality alone can select for high rates of HGT, allowing HGT to act as a hedge against uncertain opponent quality.

In our experimental system, we were only able to observe examples of T6SS cluster replacement. However, bioinformatic and phylogenetic approaches have recently identified lineages of *V. cholerae* that appear to have undergone multiple rounds of additive HGT at a single highly recombinogenic T6SS locus to form “immunity gene arrays” encoding several putative T6SS immunity proteins, which may provide protection against effectors from multiple *V. cholerae* and non-*V. cholerae* strains (16). Successive acquisition of the immunity genes likely benefits the transforming lineage in three ways. First, a recipient strain gains immunity against a diverse set of T6SS^+^ competitors. Second, the recipient avoids the metabolic cost of expressing nonnative effectors. Third, retention of the existing effector-immunity pair (as opposed to the replacement observed here) avoids costs of intrastrain genetic conflict. Thus, additive recombination represents an additional mechanism to promote horizontal spread of T6SS alleles while mitigating the costs of HGT.

In toxicogenic *V. cholerae* strains C6706 and A1552, both natural transformation and T6S are coinduced under conditions of chitin utilization, starvation, and quorum sensing at high cell density (14, 15). Specifically, activation of the quorum sensing system by *Vibrio*-specific autoinducer molecules induces the DNA uptake machinery (29, 30). This concordance suggests that *V. cholerae* may have evolved to acquire genes, including T6S effector-immunity pairs, from recently killed competitors. This has, until now, presented a conundrum: why acquire genes from defeated, inferior competitors? While it is unclear whether the bulk of horizontal transfer events occur during active T6-mediated competition, our work demonstrates that the acquisition of novel T6SSs can provide an efficient route to means-test novel weaponry, which may be strongly favored by natural selection if competitor T6S efficacy varies widely through time. More generally, our results suggest that the interplay of antagonism and horizontal transfer results in successive rounds of combat, arms trading, and selective sweeps that may dynamically shape diverse microbial communities.

## MATERIALS AND METHODS

### Bacterial strains and culture conditions.

All *V. cholerae* strains were derivatives of El Tor C6706 *str-2* (31) or environmental strain 692-79 (see Table S1 in the supplemental material). Bacteria were routinely grown at 37°C in lysogeny broth (LB) under constant shaking or statically on LB agar, supplemented with ampicillin (100 µg/ml), kanamycin (50 µg/ml), chloramphenicol (10 µg/ml for *V. cholerae* and 25 µg/ml for *Escherichia coli*), spectinomycin (100 µg/ml), streptomycin (5 mg/ml), and diaminopimelic acid (50 µg/ml) where appropriate.

10.1128/mBio.00654-17.4 TABLE S1  List of strains and plasmids used in this study. The genotype or description of each relevant *V. cholerae* strain and plasmid. Download TABLE S1, DOCX file, 0.01 MB. Copyright © 2017 Thomas et al. 2017 Thomas et al. This content is distributed under the terms of the Creative Commons Attribution 4.0 International license.

### Recombinant DNA techniques and construction of *V. cholerae* mutants.

In-frame deletions and promoter-replacement mutants in *V. cholerae* were constructed by previously described allelic exchange methods (32). Standard molecular biology-based methods were utilized for DNA manipulations. DNA-modifying enzymes and restriction nucleases (Promega and New England Biolabs); Gibson assembly mix (New England Biolabs); and Q5, Phusion, and OneTaq DNA polymerases (New England Biolabs) were used according to the manufacturer’s instructions. All recombinant DNA constructs were tested by PCR and verified by Sanger sequencing (Eurofins).

### Transformation assays.

Assays for natural transformation were performed as previously described (33). Briefly, the recipient strain was back diluted from an overnight liquid culture and grown at 37°C in LB with shaking until it reached an optical density at 600 nm (OD_600_) of 0.4 to 0.6. Cultures were washed in artificial seawater (ASW) and diluted to an OD_600_ of 0.15, and 2-ml cultures in ASW were incubated at 30°C with a chitin tile for 24 h. The chitin tile with adherent bacteria was moved to 2 ml fresh ASW containing 1 µg/ml of genomic DNA marked with an antibiotic resistance cassette and incubated at 30°C for a further 24 h. Cells were collected by washing the chitin tile in 2 ml of fresh ASW, and dilutions were plated on selective medium to determine transformation frequency. Acquisition of T6SS alleles from donor DNA linked to the antibiotic resistance cassette was verified by allele-specific multiplex PCR using primers specific for C6706 or 697-79. Transformation in coculture was performed as described above with the following exceptions: both donor and recipient strain were each grown to an OD_600_ of 0.4 to 0.6 in LB and diluted to an OD_600_ of 0.15 after washing in ASW mixed in a 1:1 ratio, and 2 ml of each mixed culture was inoculated with a chitin tile at 30°C. No exogenous genomic DNA was added.

### Killing assays.

T6SS killing assays were performed as previously described (15). Killer and prey strains grown overnight in LB at 37°C were centrifuged and resuspended in fresh medium to an OD_600_ of 1.00. Killer and prey strains were mixed at a ratio of 10:1, and 50 µl of each suspension was spotted onto a gridded filter disc (Whatman) placed on an LB agar plate and confined to 9 grid squares (3.1 by 3.1 mm each) to reduce variability. Filters were incubated at 37°C for 3 h and washed with 5 ml LB to dislodge cells, and dilutions were plated on selective medium to determine prey survival.

### Pairwise competitions.

Competitor strains were grown overnight in LB at 37°C, centrifuged, and resuspended in fresh medium to an OD_600_ of 1.00. Pairs of strains were mixed 1:1, and 50 µl of each suspension was inoculated on LB agar plugs in a 12-well plate, dried under laminar airflow in a biosafety cabinet, and incubated overnight at 37°C. Cells were resuspended in 1 ml LB, and dilutions were plated on selective medium to determine survival of each competitor. Competitive index was determined as final ratio of survivors/initial ratio of inoculation.

### Statistical analysis.

All statistics were calculated in JMP 12.0. T6SS efficacy (Fig. 2A to D) was calculated by ANOVA, followed by Tukey’s honestly significant difference (HSD) *post hoc* test, with an α of 0.05. Within each experiment (i.e., figure panel), different letters denote significantly different means. The consequence of horizontal acquisition of T6SS effectors for pairwise interactions (Fig. 3) was examined by two-tailed one-sample *t* tests (i.e., testing the null hypothesis that the competitive index is 1) with a Bonferroni correction to control for multiple comparisons. Competitive indices were log_10_ transformed prior to analysis to give equal weight to competitive ratios both above and below 1.

### Individual-based simulation.

We extended the individual-based model from the work of McNally et al. (19). Square lattices (200 × 200) were seeded with an equal number of “recipient” and “competitor” genotype cells. Biologically, this is analogous to a population growing across a solid surface, such as a crab shell. The starting conditions for Fig. 4 are shown as insets; all other simulations use the well-separated population shown in Fig. 4A. At each time point, 5% of the cells in the population were selected to reproduce if an adjacent space was unoccupied. Similarly, 5% of the cells in the population were selected to activate their T6SS. The probability that they killed a neighboring cell depended on the difference between their T6SS effector-immunity pairs and the relative killing efficiency of those effectors. The three effectors possessed by each genotype were assumed to act in an additive manner, and so the probability of killing was determined by summing across the number of effector-immunity pairs that differed between the strains, weighted by the efficacy of those effector alleles. For example, a matchup in which the recipient is inferior might be the following: the recipient genotype has three effectors, each of which confers an 0.11 probability of killing a susceptible competitor genotype cell, while the competitor has three different effectors, each of which offers an 0.33 probability of killing a susceptible recipient genotype cell. A recipient genotype cell would therefore have a 33% chance of killing an adjacent competitor cell, while the competitor would have a 100% chance of killing an adjacent recipient cell.

Recipient genotype cells had a probability, ranging from 0 to 0.005, of replacing one of their T6SS effector protein-immunity pair alleles with one obtained from the environment at each time step. This was performed in a weighted manner, according to the frequency of recipient versus competitor alleles in the population, and did not depend on the spatial distribution of those cells in the population. Biologically, this is analogous to cells sampling DNA from a well-mixed bulk medium above a surface-attached population. Note that this means that HGT can occur in both directions, with the recipient genotype replacing its T6SS with those from the competitor and, in future HGT events, reverting to its original T6SS genotype. HGT events occur in a stepwise manner, and so only one allele can be replaced per time point. The probability that adjacent cells succeed in killing was calculated as the sum of the per-allele killing probability for all of the T6SS loci that differ between the cells. Simulations were run in Python (code available upon request). Edges (outermost rows/columns) were trimmed prior to analysis. For Fig. 4 and Movies S2 and S3, fitnesses were calculated as the ratio of recipient to genotype cells at time point *t*, divided by their starting ratio. The fitness landscapes shown in these figures were calculated across a 20-by-20 factorial combination of HGT rates and competitor T6SS efficacies for 15,000 time points.

10.1128/mBio.00654-17.6 MOVIE S2 Time-lapse video of the fitness landscape shown in Fig. 4A. Download MOVIE S2, MOV file, 9.9 MB. Copyright © 2017 Thomas et al. 2017 Thomas et al. This content is distributed under the terms of the Creative Commons Attribution 4.0 International license.

10.1128/mBio.00654-17.7 MOVIE S3  Time-lapse video of the fitness landscape shown in Fig. 4B. Download MOVIE S3, MOV file, 8.4 MB. Copyright © 2017 Thomas et al. 2017 Thomas et al. This content is distributed under the terms of the Creative Commons Attribution 4.0 International license.

### Assessing fitness with variable competitors.

We examined whether or not HGT could act as a hedge against unpredictable competitors by simulating pairs of interactions, in which the T6SS killing efficacy of the recipient strain varied symmetrically (i.e., ±9.1, 27.2, 36.4, or 45.5% above and below) around the competitor’s killing efficacy. We calculated the geometric mean of fitness (raw fitness calculated as described above) for these pairs of interactions across a range of HGT rates (0 to 0.005), with five replicates of the simulation run per treatment combination.

## References

[1] Soucy SM, Huang J, Gogarten JP 2015 Horizontal gene transfer: building the web of life. Nat Rev Genet 16:472–482. doi:10.1038/nrg3962. 26184597

[2] Chun J, Grim CJ, Hasan NA, Lee JH, Choi SY, Haley BJ, Taviani E, Jeon YS, Kim DW, Lee JH, Brettin TS, Bruce DC, Challacombe JF, Detter JC, Han CS, Munk AC, Chertkov O, Meincke L, Saunders E, Walters RA, Huq A, Nair GB, Colwell RR 2009 Comparative genomics reveals mechanism for short-term and long-term clonal transitions in pandemic *Vibrio cholerae*. Proc Natl Acad Sci U S A 106:15442–15447. doi:10.1073/pnas.0907787106. 19720995 PMC2741270

[3] Katz LS, Petkau A, Beaulaurier J, Tyler S, Antonova ES, Turnsek MA, Guo Y, Wang S, Paxinos EE, Orata F, Gladney LM, Stroika S, Folster JP, Rowe L, Freeman MM, Knox N, Frace M, Boncy J, Graham M, Hammer BK, Boucher Y, Bashir A, Hanage WP, Van Domselaar G, Tarr CL 2013 Evolutionary dynamics of *Vibrio cholerae* O1 following a single-source introduction to Haiti. mBio 4:e00398-13. doi:10.1128/mBio.00398-13. 23820394 PMC3705451

[4] Meibom KL, Blokesch M, Dolganov NA, Wu CY, Schoolnik GK 2005 Chitin induces natural competence in *Vibrio cholerae*. Science 310:1824–1827. doi:10.1126/science.1120096. 16357262

[5] Waldor MK, Mekalanos JJ 1996 Lysogenic conversion by a filamentous phage encoding cholera toxin. Science 272:1910–1914. doi:10.1126/science.272.5270.1910. 8658163

[6] Pruzzo C, Vezzulli L, Colwell RR 2008 Global impact of *Vibrio cholerae* interactions with chitin. Environ Microbiol 10:1400–1410. doi:10.1111/j.1462-2920.2007.01559.x. 18312392

[7] Alcoforado Diniz J, Liu YC, Coulthurst SJ 2015 Molecular weaponry: diverse effectors delivered by the type VI secretion system. Cell Microbiol 17:1742–1751. doi:10.1111/cmi.12532. 26432982 PMC4832377

[8] Durand E, Cambillau C, Cascales E, Journet L 2014 VgrG, Tae, Tle, and beyond: the versatile arsenal of type VI secretion effectors. Trends Microbiol 22:498–507. doi:10.1016/j.tim.2014.06.004. 25042941

[9] Joshi A, Kostiuk B, Rogers A, Teschler J, Pukatzki S, Yildiz FH 2017 Rules of engagement: the type VI secretion system in *Vibrio cholerae*. Trends Microbiol 25:267–279. doi:10.1016/j.tim.2016.12.003. 28027803 PMC5365375

[10] Russell AB, Peterson SB, Mougous JD 2014 Type VI secretion system effectors: poisons with a purpose. Nat Rev Microbiol 12:137–148. doi:10.1038/nrmicro3185. 24384601 PMC4256078

[11] Cianfanelli FR, Monlezun L, Coulthurst SJ 2016 Aim, load, fire: the type VI secretion system, a bacterial nanoweapon. Trends Microbiol 24:51–62. doi:10.1016/j.tim.2015.10.005. 26549582

[12] Bernardy EE, Turnsek MA, Wilson SK, Tarr CL, Hammer BK 2016 Diversity of clinical and environmental isolates of *Vibrio cholerae* in natural transformation and contact-dependent bacterial killing indicative of type VI secretion system activity. Appl Environ Microbiol 82:2833–2842. doi:10.1128/AEM.00351-16. 26944842 PMC4836410

[13] Ishikawa T, Sabharwal D, Bröms J, Milton DL, Sjöstedt A, Uhlin BE, Wai SN 2012 Pathoadaptive conditional regulation of the type VI secretion system in *Vibrio cholerae* O1 strains. Infect Immun 80:575–584. doi:10.1128/IAI.05510-11. 22083711 PMC3264300

[14] Borgeaud S, Metzger LC, Scrignari T, Blokesch M 2015 The type VI secretion system of *Vibrio cholerae* fosters horizontal gene transfer. Science 347:63–67. doi:10.1126/science.1260064. 25554784

[15] Watve SS, Thomas J, Hammer BK 2015 CytR is a global positive regulator of competence, type VI secretion, and chitinases in *Vibrio cholerae*. PLoS One 10:e0138834. doi:10.1371/journal.pone.0138834. 26401962 PMC4581735

[16] Kirchberger PC, Unterweger D, Provenzano D, Pukatzki S, Boucher Y 2017 Sequential displacement of type VI secretion system effector genes leads to evolution of diverse immunity gene arrays in *Vibrio cholerae*. Sci Rep 7:45133. doi:10.1038/srep45133. 28327641 PMC5361080

[17] Salomon D, Klimko JA, Trudgian DC, Kinch LN, Grishin NV, Mirzaei H, Orth K 2015 Type VI secretion system toxins horizontally shared between marine bacteria. PLoS Pathog 11:e1005128. doi:10.1371/journal.ppat.1005128. 26305100 PMC4549250

[18] Unterweger D, Miyata ST, Bachmann V, Brooks TM, Mullins T, Kostiuk B, Provenzano D, Pukatzki S 2014 The *Vibrio cholerae* type VI secretion system employs diverse effector modules for intraspecific competition. Nat Commun 5:3549. doi:10.1038/ncomms4549. 24686479 PMC3988814

[19] McNally L, Bernardy E, Thomas J, Kalziqi A, Pentz J, Brown SP, Hammer BK, Yunker PJ, Ratcliff WC 2017 Killing by type VI secretion drives genetic phase separation and correlates with increased cooperation. Nat Commun 8:14371. doi:10.1038/ncomms14371. 28165005 PMC5303878

[20] Watve SS, Chande AT, Rishishwar L, Mariño-Ramírez L, Jordan IK, Hammer BK 2016 Whole-genome sequences of 26 *Vibrio cholerae* isolates. Genome Announc 4:e01396-16. doi:10.1128/genomeA.01396-16. PMC5180380 28007852

[21] Altindis E, Dong T, Catalano C, Mekalanos J 2015 Secretome analysis of *Vibrio cholerae* type VI secretion system reveals a new effector-immunity pair. mBio 6:e00075-15. doi:10.1128/mBio.00075-15. 25759499 PMC4453574

[22] Unterweger D, Kostiuk B, Pukatzki S 2017 Adaptor proteins of type VI secretion system effectors. Trends Microbiol 25:8–10. doi:10.1016/j.tim.2016.10.003. 27856117

[23] Dong TG, Ho BT, Yoder-Himes DR, Mekalanos JJ 2013 Identification of T6SS-dependent effector and immunity proteins by Tn-seq in *Vibrio cholerae*. Proc Natl Acad Sci U S A 110:2623–2628. doi:10.1073/pnas.1222783110. 23362380 PMC3574944

[24] Russell AB, LeRoux M, Hathazi K, Agnello DM, Ishikawa T, Wiggins PA, Wai SN, Mougous JD 2013 Diverse type VI secretion phospholipases are functionally plastic antibacterial effectors. Nature 496:508–512. doi:10.1038/nature12074. 23552891 PMC3652678

[25] Brooks TM, Unterweger D, Bachmann V, Kostiuk B, Pukatzki S 2013 Lytic activity of the *Vibrio cholerae* type VI secretion toxin VgrG-3 is inhibited by the antitoxin TsaB. J Biol Chem 288:7618–7625. doi:10.1074/jbc.M112.436725. 23341465 PMC3597803

[26] Miyata ST, Kitaoka M, Brooks TM, McAuley SB, Pukatzki S 2011 *Vibrio cholerae* requires the type VI secretion system virulence factor VasX to kill *Dictyostelium discoideum*. Infect Immun 79:2941–2949. doi:10.1128/IAI.01266-10. 21555399 PMC3191968

[27] Ma LS, Lin JS, Lai EM 2009 An IcmF family protein, ImpLM, is an integral inner membrane protein interacting with ImpKL, and its Walker A motif is required for type VI secretion system-mediated Hcp secretion in *Agrobacterium tumefaciens*. J Bacteriol 191:4316–4329. doi:10.1128/JB.00029-09. 19395482 PMC2698499

[28] Simons AM 2009 Fluctuating natural selection accounts for the evolution of diversification bet hedging. Proc Biol Sci 276:1987–1992. doi:10.1098/rspb.2008.1920. 19324774 PMC2677257

[29] Antonova ES, Hammer BK 2011 Quorum-sensing autoinducer molecules produced by members of a multispecies biofilm promote horizontal gene transfer to *Vibrio cholerae*. FEMS Microbiol Lett 322:68–76. doi:10.1111/j.1574-6968.2011.02328.x. 21658103

[30] Suckow G, Seitz P, Blokesch M 2011 Quorum sensing contributes to natural transformation of *Vibrio cholerae* in a species-specific manner. J Bacteriol 193:4914–4924. doi:10.1128/JB.05396-11. 21784943 PMC3165701

[31] Thelin KH, Taylor RK 1996 Toxin-coregulated pilus, but not mannose-sensitive hemagglutinin, is required for colonization by *Vibrio cholerae* O1 El Tor biotype and O139 strains. Infect Immun 64:2853–2856. 8698524 10.1128/iai.64.7.2853-2856.1996 PMC174155

[32] Skorupski K, Taylor RK 1996 Positive selection vectors for allelic exchange. Gene 169:47–52. doi:10.1016/0378-1119(95)00793-8. 8635748

[33] Watve SS, Bernardy EE, Hammer BK 2014 Vibrio cholerae: measuring natural transformation frequency. Curr Protoc Microbiol 35:6A.4.1–6A.412. doi:10.1002/9780471729259.mc06a04s35. 25367272

