# Supplementary figures and images for: Horizontal Gene Transfer of Functional Type VI Killing Genes by Natural Transformation
Source: mBio. 2017 Jul 25;8(4):e00654-17. doi: 10.1128/mBio.00654-17 (PMC5527308; doi:10.1128/mBio.00654-17)

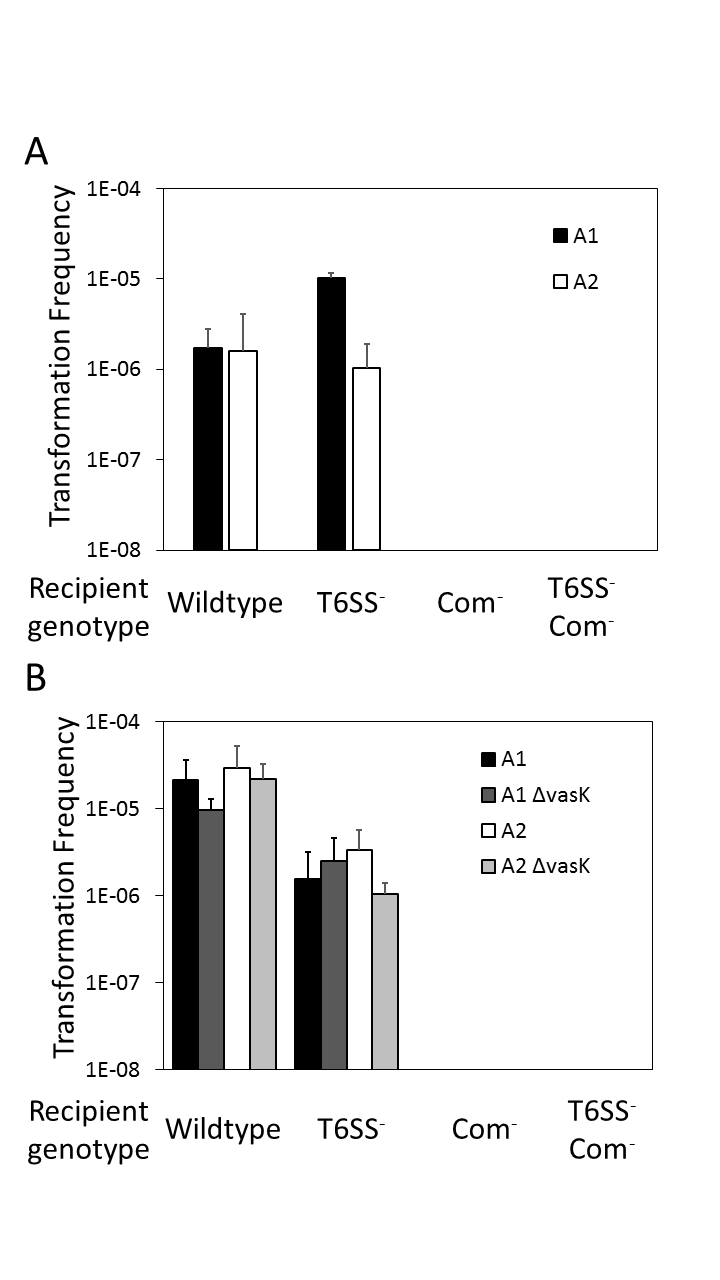

Supplement: FIG S1 [file mbo001173396sf1.tif]

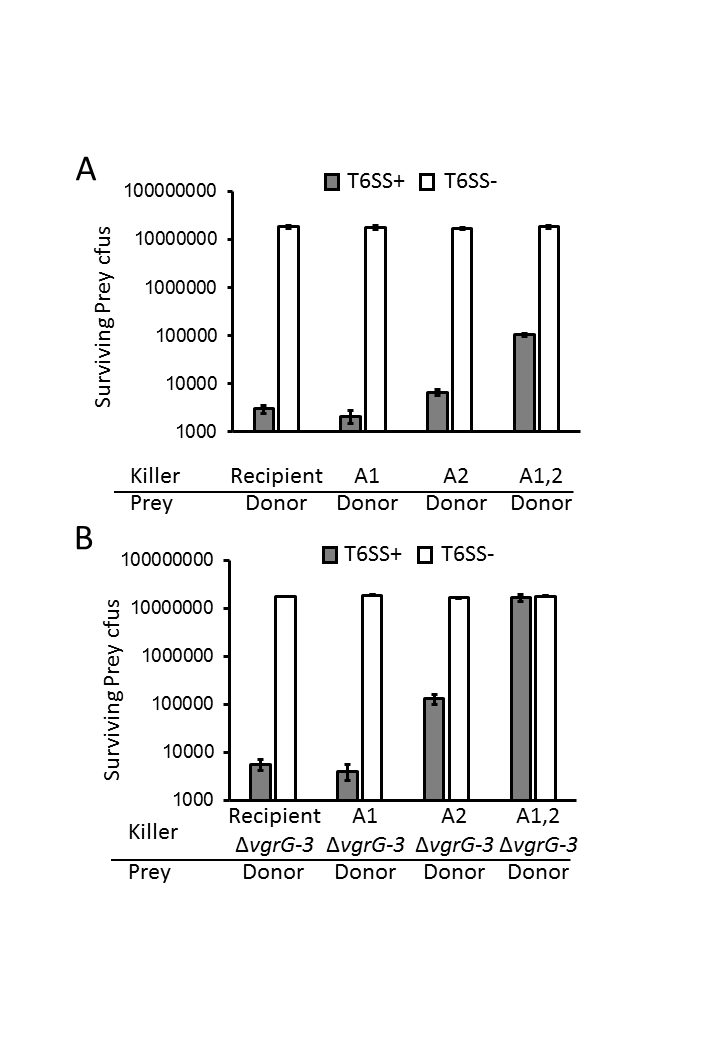

Supplement: FIG S2 [file mbo001173396sf2.tif]

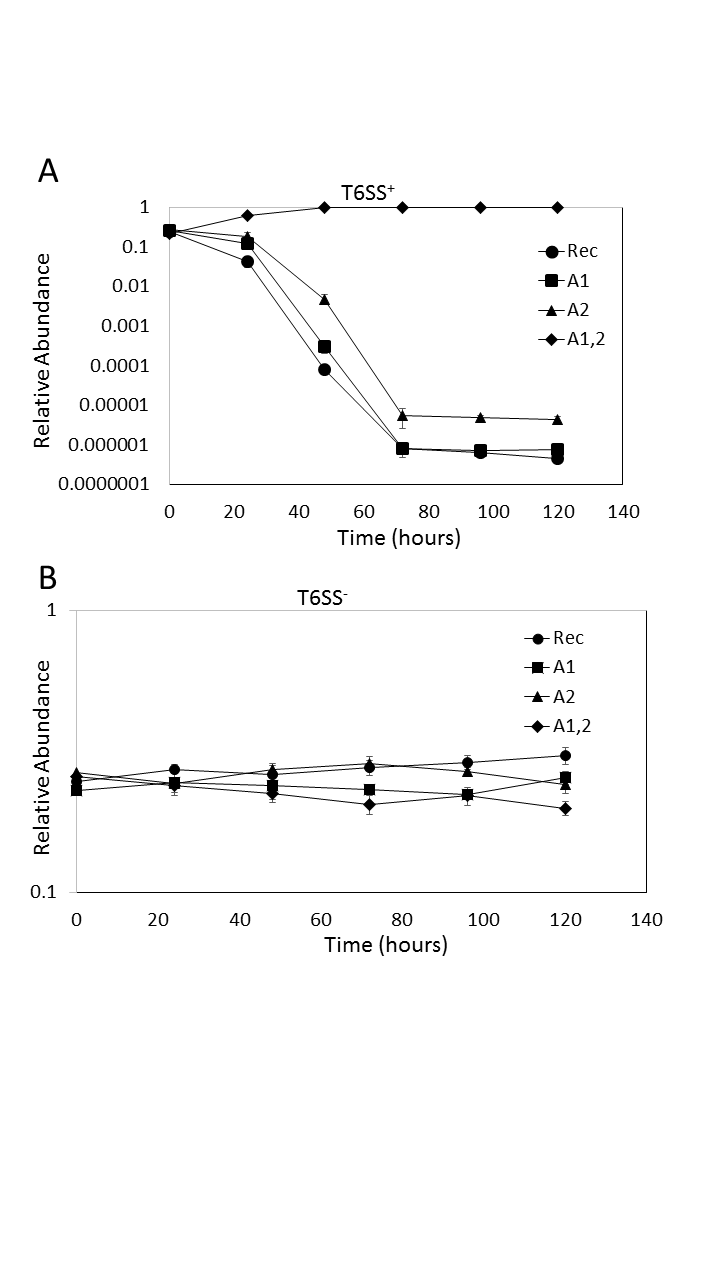

Supplement: FIG S3 [file mbo001173396sf3.tif]
