# Supplementary material for: Horizontal Gene Transfer of Functional Type VI Killing Genes by Natural Transformation
Source: mBio. 2017 Jul 25;8(4):e00654-17. doi: 10.1128/mBio.00654-17 (PMC5527308; doi:10.1128/mBio.00654-17)
Supplement: TABLE S1 [file mbo001173396st1.docx]

**Table S1.** List of strains and plasmids used in this study.

| **Strains** | **Genotype or Description** | **Reference** |
| --- | --- | --- |
| BH1514 | C6706 El Tor *str-2* | K. H. Thelin and R. K. Taylor. *Infect. Immun.* 64(7): 2853-2856, 1996. |
| 692-79 | Environmental isolate | E. E. Bernardy *et al.* *Appl. Environ. Microbiol.* 82(9): 2833-3842, 2016. |
| JT946 | 692-79 Δ*vasK* | L. McNally *et al*. *Nat. Commun.* 8, 14371 doi: 10.1038/ncomms14371, 2017. |
| JT966 | 692-79 *lacZ*::Kan^R^, *mKO* | This study |
| JT967 | 692-79 Δ*vasK* *lacZ*::Kan^R^, *mKO* | This study |
| JT1035 | 692-79 *vc1420*::pJT1033 | This study |
| JT1036 | 692-79 *vca0022*::pJT1034 | This study |
| JT1031 | C6706 *ptac-qstR* Δ*vc1807ΩmKO* Δ*lacZΩ*Spec^R^ | This study |
| JT1032 | C6706 *ptac-qstR* Δ*vasK* Δ*vc1807ΩmKO* Δ*lacZΩ*Spec^R^ | This study |
| SW638 | C6706 *ptac-qstR* Δ*vc1807ΩmKO* Δ*lacZΩ*Kan^R^ |  |
| SW639 | C6706 *ptac-qstR* Δ*vasK* Δ*vc1807ΩmKO* Δ*lacZΩ*Kan^R^ | This study |
| JT1040 | C6706 *ptac-qstR* Δ*vc1807ΩmKO* Δ*lacZΩ*Spec^R^ T6SS Aux-1 replaced with 692-79 Aux-1 | This study |
| JT1041 | C6706 *ptac-qstR* Δ*vasK* Δ*vc1807ΩmKO* Δ*lacZΩ*Spec^R^ T6SS Aux-1 replaced with 692-79 Aux-1 | This study |
| JT1110 | C6706 *ptac-qstR* Δ*vc1807ΩmKO* Δ*lacZΩ*Kan^R^ T6SS Aux-1 replaced with 692-79 Aux-1 | This study |
| JT1111 | C6706 *ptac-qstR* Δ*vasK* Δ*vc1807ΩmKO* Δ*lacZΩ*Kan^R^ T6SS Aux-1 replaced with 692-79 Aux-1 | This study |
| SW632 | C6706 *ptac-qstR* Δ*vc1807ΩmKO* Δ*lacZΩ*Spec^R^ T6SS Aux-2 replaced with 692-79 Aux-2 | This study |
| SW642 | C6706 *ptac-qstR* Δ*vasK* Δ*vc1807ΩmKO* Δ*lacZΩ*Spec^R^ T6SS Aux-2 replaced with 692-79 Aux-2 | This study |
| SW643 | C6706 *ptac-qstR* Δ*vc1807ΩmKO* Δ*lacZΩ*Amp^R^ T6SS Aux-1 replaced with 692-79 Aux-2 | This study |
| SW644 | C6706 *ptac-qstR* Δ*vasK* Δ*vc1807ΩmKO* Δ*lacZΩ*Amp^R^ T6SS Aux-1 replaced with 692-79 Aux-2 | This study |
| JT1045 | C6706 *ptac-qstR* Δ*vc1807ΩmKO* Δ*lacZΩ*Spec^R^ T6SS Aux-1&2 replaced with 692-79 Aux-1&2 | This study |
| JT1046 | C6706 *ptac-qstR* Δ*vasK* Δ*vc1807ΩmKO* Δ*lacZΩ*Spec^R^ T6SS Aux-1&2 replaced with 692-79 Aux-1&2 | This study |
| JT1054 | C6706 *ptac-qstR* Δ*vc1807ΩmKO* Δ*lacZΩ*Cm^R^ T6SS Aux-1&2 replaced with 692-79 Aux-1&2 | This study |
| JT1055 | C6706 *ptac-qstR* Δ*vasK* Δ*vc1807ΩmKO* Δ*lacZΩ*Cm^R^ T6SS Aux-1&2 replaced with 692-79 Aux-1&2 | This study |
| JT1102 | C6706 *ptac-qstR* Δ*vc1807ΩmKO* Δ*lacZΩ*Spec^R^ Δ*vgrG-3* | This study |
| JT1103 | C6706 *ptac-qstR* Δ*vasK* Δ*vc1807ΩmKO* Δ*lacZΩ*Spec^R^ Δ*vgrG-3* | This study |
| JT1104 | C6706 *ptac-qstR* Δ*vc1807ΩmKO* Δ*lacZΩ*Spec^R^ T6SS Aux-1 replaced with 692-79 Aux-1 Δ*vgrG-3* | This study |
| JT1105 | C6706 *ptac-qstR* Δ*vasK* Δ*vc1807ΩmKO* Δ*lacZΩ*Spec^R^ T6SS Aux-1 replaced with 692-79 Aux-1 Δ*vgrG-3* | This study |
| JT1106 | C6706 *ptac-qstR* Δ*vc1807ΩmKO* Δ*lacZΩ*Spec^R^ T6SS Aux-2 replaced with 692-79 Aux-2 Δ*vgrG-3* | This study |
| JT1107 | C6706 *ptac-qstR* Δ*vasK* Δ*vc1807ΩmKO* Δ*lacZΩ*Spec^R^ T6SS Aux-2 replaced with 692-79 Aux-2 Δ*vgrG-3* | This study |
| JT1108 | C6706 *ptac-qstR* Δ*vc1807ΩmKO* Δ*lacZΩ*Spec^R^ T6SS Aux-1&2 replaced with 692-79 Aux-1&2 Δ*vgrG-3* | This study |
| JT1109 | C6706 *ptac-qstR* Δ*vasK* Δ*vc1807ΩmKO* Δ*lacZΩ*Spec^R^ T6SS Aux-1&2 replaced with 692-79 Aux-1&2 Δ*vgrG-3* | This study |
| **Plasmids** | **Features** | **Reference** |
| pKAS32 | Allelic exchange vector | K. Skorupski and R. K. Taylor, *Gene*. 169(1): 47-52, 1996 |
| pJT961 | Allelic exchange vector, *aph* cassette cloned in BpmI-ScaI sites of pKAS32, replacing internal fragment of *bla*. Amp^S^ Kan^R^ | This study |
| pJT1033 | 500 bp downstream sequence of T6SS Aux1 of BGT64 cloned in EcoRI-XbaI sites of pJT961, Kan^R^ | This study |
| pJT1034 | 500 bp downstream sequence of T6SS Aux2 of BGT64 cloned in EcoRI-XbaI sites of pJT961, Kan^R^ | This study |
